# Long-term stability of cellulose acetate butyrate thin films for nuclear certified reference materials

**DOI:** 10.1007/s10967-016-5083-z

**Published:** 2016-10-27

**Authors:** Renáta Buják, Laurens Delva, Mustafa Erkoç, Jeroen Bauwens, Rožle Jakopič, Laszlo Vincze, Yetunde Aregbe, Ludwig Cardon

**Affiliations:** 1 Directorate G: Nuclear Safety & Security, Joint Research Centre, European Commission, Retieseweg 111, 2440 Geel, Belgium; 2 Centre for Polymer & Material Technologies (CPMT), Faculty of Engineering and Architecture, Ghent University, Technologiepark 915, 9052 Zwijnaarde, Belgium; 3 Department of Analytical Chemistry, Faculty of Sciences, Ghent University, Krijgslaan 281, S12, 9000 Ghent, Belgium

**Keywords:** Nuclear safeguards, Cellulose acetate butyrate, Ageing, Large-sized dried spike, Uranium, Plutonium

## Abstract

Characterization of cellulose acetate butyrate (CAB) thin films with 17, 35 and 52 wt% butyryl is carried out to select the most suitable matrix material for the U and Pu containing large-sized dried spike reference material. The virgin CAB samples were aged by vibrations, heat, humidity, UV light and X-rays. Characterization was done by thermo-analytical techniques, gel permeation chromatography, mechanical tests and via Rayleigh and Compton scattering. The results show that CAB with lower butyryl content can withstand higher operational temperatures and has greater mechanical strength while CAB with higher butyryl content seems to be more resistant to radiation.

## Introduction

Large-sized dried spike containing uranium and plutonium are essential part of the nuclear material accountancy for safeguards purposes. The Treaty on Non-Proliferation of Nuclear Weapons and the EURATOM Treaty require the signatory states to provide detailed accounting records for their fissile materials [1, 2]. The aim of nuclear safeguards is the verification of fissile material for its intended and declared peaceful use. This involves accurate measurements of uranium and plutonium amount content and isotopic composition of spent fuel solution at reprocessing plants. The IRMM-1027 large-sized dried (LSD) spikes are tailor-made certified reference materials (CRMs) that are designed for such purposes [3]. The LSD spikes are produced exclusively by JRC-Geel and the International Atomic Energy Agency in Vienna. The production is on a small laboratory scale at the JRC-Geel site and every year 1200 units are prepared. The starting materials are high purity U and Pu metals, which are either natural (U) or isotopically enriched (U, Pu) [4]. Each individual spike contains about 50 mg uranium (*m*(^235^U)/*m*(U) ~ 20 %) and 1.8 mg plutonium (*m*(^239^Pu)/*m*(Pu) ~ 98 %) in dried nitrate form, allowing the undiluted spent fuel solution to be added quantitatively to them. The measurements are carried out with Isotope Dilution Thermal Ionization Mass Spectrometry (ID-TIMS) by plant operators and safeguards authorities by the European Commission and also outside EU [5]. The uncertainty that is introduced by this reference material is very low, and allows the laboratories to achieve IDMS measurements results with uncertainties below the target value of 0.28 % for hot-cell conditions, expressed as a relative standard uncertainty [6, 7].

**Fig. 1:**
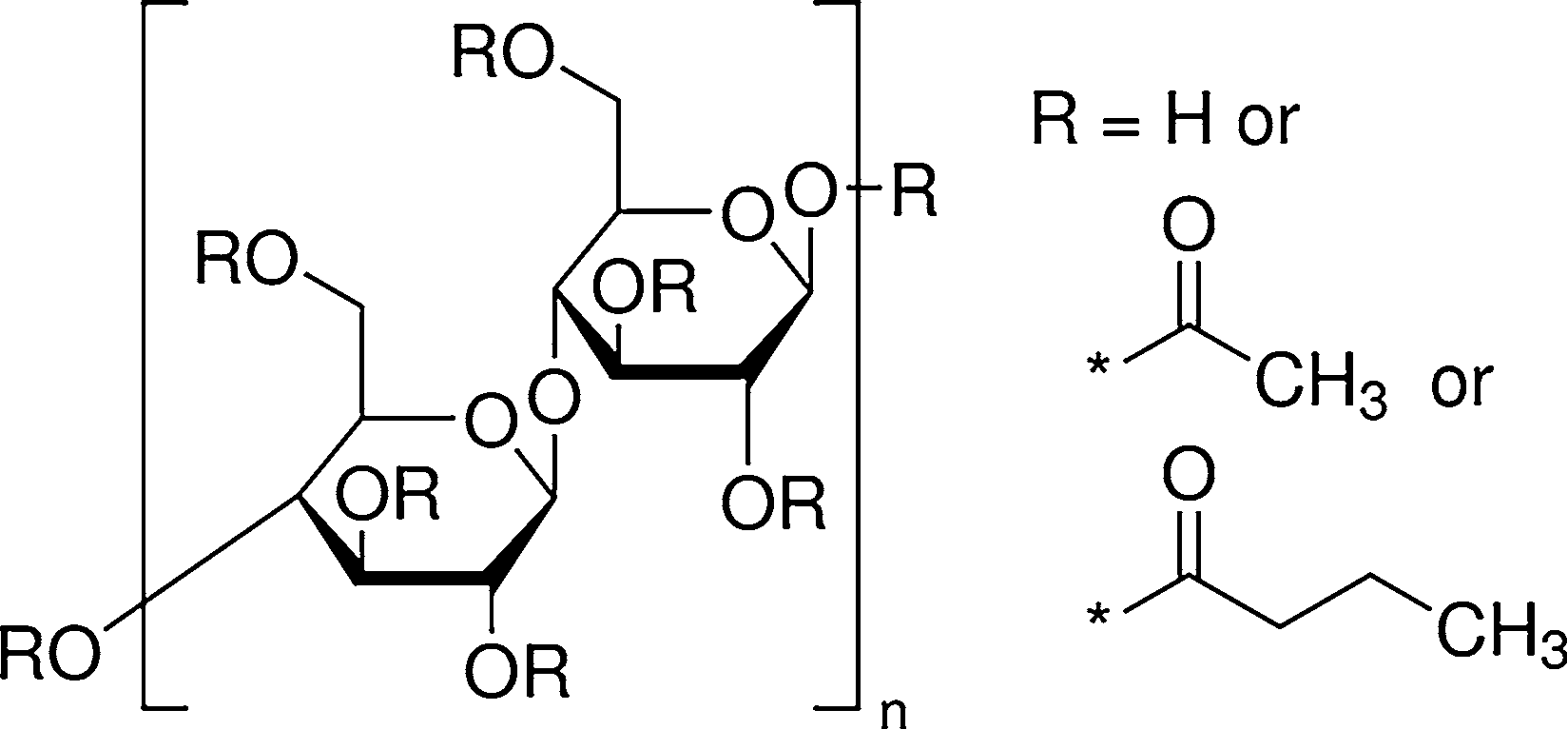
Chemical structure of cellulose acetate butyrate [12]

In the past, batches of LSD spikes were used within a short period of time, approximately half a year or 1 year. The demand of the market however has been changed during the last decade. Additionally, the availability of the precious and scarce high purity and enriched Pu-239 reference metal is limited, so the long term stability of the LSD spikes became crucial for the producer as well as for the customers. The IRMM-1027 series of LSD spikes are prepared in penicillin glass vials to allow them to be gripped and moved with manipulators inside the hot-cells. For storage and transport purposes, the solid uranyl and plutonium nitrate are fixed at the bottom of the vial with an organic coating, which serves also as a supporting matrix for the spikes. There are stringent requirements for this coating material deposited onto the U/Pu solid spikes: it should be readily dissolved in hot HNO_3_; it should preferentially be an organic compound without halogens; it should be resistant to radiolysis—or at least should not degrade within the timeframe of the validity of the certificate; it should adhere well to glass; it should be easy to handle and dispense; it should not cause interference with the IDMS measurements and it should have sufficient mechanical strength. During the 1990s, tetrahydrofuran (THF) was applied as coating which formed stable complexes with U and Pu but it required a long and complex dissolution procedure [8]. Customers’ feedback at the time showed that they preferred a spike that could be easily dissolved and used. Cellulose acetate butyrate (CAB) was therefore chosen as a ‘LSD producer-’ and ‘LSD customer’-friendly alternative for THF [9]. Cellulose esters are widley use in the industy as coatings, plastics, membranes [10], and particularly CAB is also used as nuclear track detector [11]. CAB is an ester of cellulose that comes with different acetyl and butyryl substitution of hydroxyl groups (see Fig. 1).

This substitution is usually expressed in wt% as approximate content of the corresponding group at the triester of the pyranose ring. The properties of CAB depend on the degree of substitution: usually the higher the butyryl content the higher the flexibility and the chemical resistance. In the literature, several studies can be found on the thermal degradation, on the mechanical properties, on the degradation due to irradiation of cellulose and cellulose derivatives [11, 13–16, 13–16] However, so far no research paper can be found which deals with the application of cellulose acetate butyrate on nuclear materials as a thin film coating aiming to understand the complex degradation behaviour caused by heat, radiation and vibrations. The main objective of this study was to characterize before and after ageing and to compare the degradation behaviour of three commercially available CABs in order to find the best appropriate butyryl substitution for the IRMM 1027 LSD spike application. CAB with 17 wt% butyryl content was successfully used on the initial investigations with metallic spikes as well as on the LSD spikes [17]. However, there are only empirical data available concerning the life time of the CAB with 17 wt% butyryl substitution which shows that this layer tends to chip and flake off after about 2 years of storage. This does not meet anymore the current demand from the end-users to extend the life time of the LSD spikes beyond 2 years. Empirical studies at JRC-Geel already showed that CAB with 35 wt% butyryl applied on U/Pu LSD spikes preserved the integrity of the spike for more than 3 years. Hence, our aim was to find the best suitable butyryl substitution of CAB for future IRMM-1027 LSD spikes that complies with all the above mentioned criteria.

In the current paper, the preparation procedure of the CAB layer on LSD spikes is presented and the thermal, chemical and mechanical properties of the virgin and aged polymers without uranyl- and Pu-nitrate were investigated. Three commercially available CABs were tested: 17–19 wt% (CAB-17) as the lowest butyryl substitution, 35–39 wt% (CAB-35) as an intermediate butyryl substitution and 52 wt% (CAB-52) as the highest available butyryl substitution. The physical and chemical degradation were investigated by carrying out transport and ageing tests. The chemical ageing was accelerated by humidity, temperature, UV and X-ray irradiation. Both the virgin and aged thin films were characterized by thermogravimetry analysis (TGA), differential scanning calorimetry (DSC), gel permeation chromatography (GPC), micro-X-ray fluorescence (XRF) and by tensile testing. The different ageing experiments were carried out independently, thus the combination of these are not part of our study. However, in reality the samples are under constant irradiation; and the exposure to variation of temperature, UV light and vibrations are happening mostly during transport.

## Experimental

CAB with 17 wt% butyryl content was purchased from Sigma-Aldrich, CAB with 35 wt% butyryl from Acrōs while CAB with 52 wt% butyryl was acquired from Eastman Chemicals. The properties of the three different CABs are shown in Table 1. The thin film samples were prepared by evaporating the solvent from the 10 wt% CAB solution prepared in acetone. There were two types of thin film samples, one type prepared in the original penicillin vial and the other in a Teflon mould. The latter was prepared by pouring and spreading the solution into the mould obtaining thin film rectangular samples after evaporation of the solvent. The dimensions of the rectangular samples were as follows: width 10 mm, thickness approximately 0.2 mm and length 150 mm.

**Table 1:** Properties of the cellulose acetate butyrate as supplied by the manufacturer

	*M*_n_ (g mol^−1^)	Butyryl content (wt%)	Acetyl content (wt%)	Hydroxyl content (wt%)
CAB-17	65,000	16.5–19.0	28.0–31.0	0.8–1.4
CAB-35	70,000	35.0–39.0	12.0–15.0	1.8
CAB-52	30,000	52.0	2.0	1.8

The rectangular samples could then be cut into the desired sample form using a scalpel. For the transport tests, Mg(NO_3_)_2_ × 6H_2_O was used as an inactive replacement salt of the U and Pu-nitrates. First, 2.5 mL solution of 2 wt% Mg(NO_3_)_2_ in 5 mol L^−1^ HNO_3_ was dispensed into the vials and evaporated. Then, 0.7 mL 10 wt% CAB solution in acetone was applied and evaporated to create a similar structure to that found in the spike material.

### Transport simulations

The samples were handled in the same manner as the real LSD spikes. After the preparation of the CAB with the magnesium nitrate salt in the penicillin vials, they were closed with a silicon cap and placed into PVC bags. The bags were sealed individually and put into a type EMMA container filled with expanded polystyrene. This container was then placed on the transport simulator. The standard used for simulating the transport by plane was the International Safe Transit Association (ISTA) air (60 min/run, 200 Hz). The total simulation consisted of real transport by car and simulations by truck and airplane. The transport simulations run for 1, 2 and 3 h. At each time, visual inspection was made to check whether the coating was intact.

### UV light irradiation

The UV xenon test was performed on a Xenon test 1200 apparatus according to ISO 4892, method A [18]. Chamber temperature and relative humidity were controlled at 38 ± 3 °C and 50 ± 10 % respectively. The test was performed using both the rectangular thin film samples and the glass vials. Both types of samples were packed and sealed in PVC bags. Samples were evaluated at 1000 h—approximating to 1 year of weathering conditions—and at 2000 h—to 2 years of weathering conditions.

### X-ray irradiation

The degradation through time of the different types of CAB was monitored by applying a focused, monochromatic 17.4 keV X-ray beam. The X-ray source was an Xbeam (X-ray Optical Systems Inc., Albany, NY, USA) microfocus tube equipped with a doubly curved crystal optic for focusing and monochromatisation. The sample of 100 × 100 μm was attached to an aluminium plate by a transparent Scotch tape. The X-ray tube was operated at 40 kV, 0.800 mA, the spot size was ≈150 (H) × 50 (V) μm^2^ full width at half maximum, single spot measurement and the Compton and Rayleigh scatter spectra were saved every 1000 s real time.

### Thermogravimetric analysis (TGA)

The TGA measurements were carried out with a Netzsch 449 F3 Jupiter type analyser. Approximately 20 mg of the samples were placed in a Pt–Rh crucible and heated up to 600 °C with a heating rate of 10 °C min^−1^ in nitrogen atmosphere. The mass changes were recorded with time and with increasing temperature. Only the virgin samples were tested.

### Differential scanning calorimetry (DSC)

DSC measurements were performed on a Netzsch DSC 204 F1. Aluminium crucibles were used and the experiments were run under an inert nitrogen atmosphere (20 mL min^−1^). A heating/cooling programme between 25 and 280 °C with a scanning rate of 10 °C min^−1^ was applied. Approximately 20 mg of sample material was used. The glass transition temperature and melting temperature were measured for the three different types of CAB thin films. Only the virgin samples were tested.

### Gel permeation chromatography (GPC)

GPC was used as a direct way to measure degradation by determining the molecular weight of the samples. GPC measurements were performed on a Waters Instrument, with RI detector (2414 Waters), equipped with three Polymer Standards Services GPC serial columns (1× GRAM Analytical 30 Å, 10 µm and 2× GRAM Analytical 1000 Å, 10 µm). Polystyrene standards were used for calibration. The CAB films were dissolved in chloroform and 20 μL solution was injected over the column with a run time of about 40 min. The following molecular weights were measured: Number-average molecular weight (*M*_n_), weight-average molecular weight (*M*_w_), *z*-average molecular weight (*M*_*Z*_), peak molecular weight (*M*_P_); and the molar mass dispersity (DM) were calculated [19]. Both the virgin (not aged) and the UV aged samples were analysed.

### Tensile test

Tensile testing was done according to ISO 527 to determine the following values: stress at break, Young’s modulus and strain at break [20]. An Instron 5565 apparatus was used and the load of the cell was 2000 N. Crosshead speed was fixed at 10 mm min^−1^. The virgin and the UV aged samples were tested.

## Results and discussion

### Production of IRMM-1027 series

A short introduction on the preparation of the LSD spikes is presented in order to better explain the sample preparation procedure and the design of the different type of experiments [21, 22]. Each year about 1200 units of IRMM-1027 series of LSD spikes are prepared to fulfil the demands for fissile material control. High purity U and Pu certified reference metals are accurately weighed and dissolved in 5 mol L^−1^ HNO_3_. Aliquots of 2.5 mL of the stock solution are dispensed into individual penicillin vials by an automated system [23]. The nitrate solution in the vial containing the U and Pu is evaporated to complete dryness. As uranyl nitrate is hygroscopic and CAB sensitive for moisture uptake, the humidity in the glove box is controlled and kept below 20–25 % relative humidity. The dried UO_2_(NO_3_)_2_ and Pu(NO_3_)_6_ are treated with a 0.7 mL 10 wt% CAB solution in acetone and kept under ventilation to let the acetone evaporate on air. As the nitrates are soluble in acetone, the final product is a layer of CAB capturing the uranyl- and plutonium nitrates. The evaporation takes about 3 h. The vials are then placed on a hotplate at 45–50 °C for 45 min to remove any solvent residues. Finally, the vials are capped and sealed in plastic bags.

### Thermal behaviour of the virgin CAB samples

**Fig. 2:**
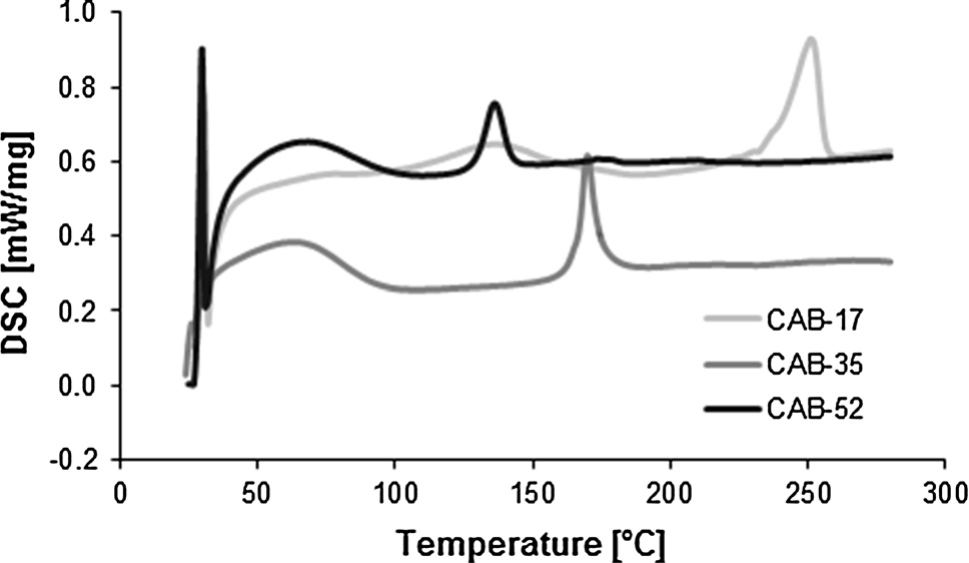
DSC curves of the virgin CAB-17, CAB-35 and CAB-52 samples

Figure 2 shows the DSC curves of the three CAB samples. Analysing the curves, it can be seen that with higher butyryl content the melting peak shifts towards lower temperatures. The same tendency can be observed in case of the glass transition temperatures as well (see Table 2). This is an indication that CAB with low butyryl content can withstand higher operational temperatures and thus is thermally more stable than one with high butyryl content.

**Table 2:** Thermal parameters from the DSC analysis

	CAB-17	CAB-35	CAB-52
Melt peak (°C)	251	170	136
Glass transition (°C)	145	129	103

**Fig. 3:**
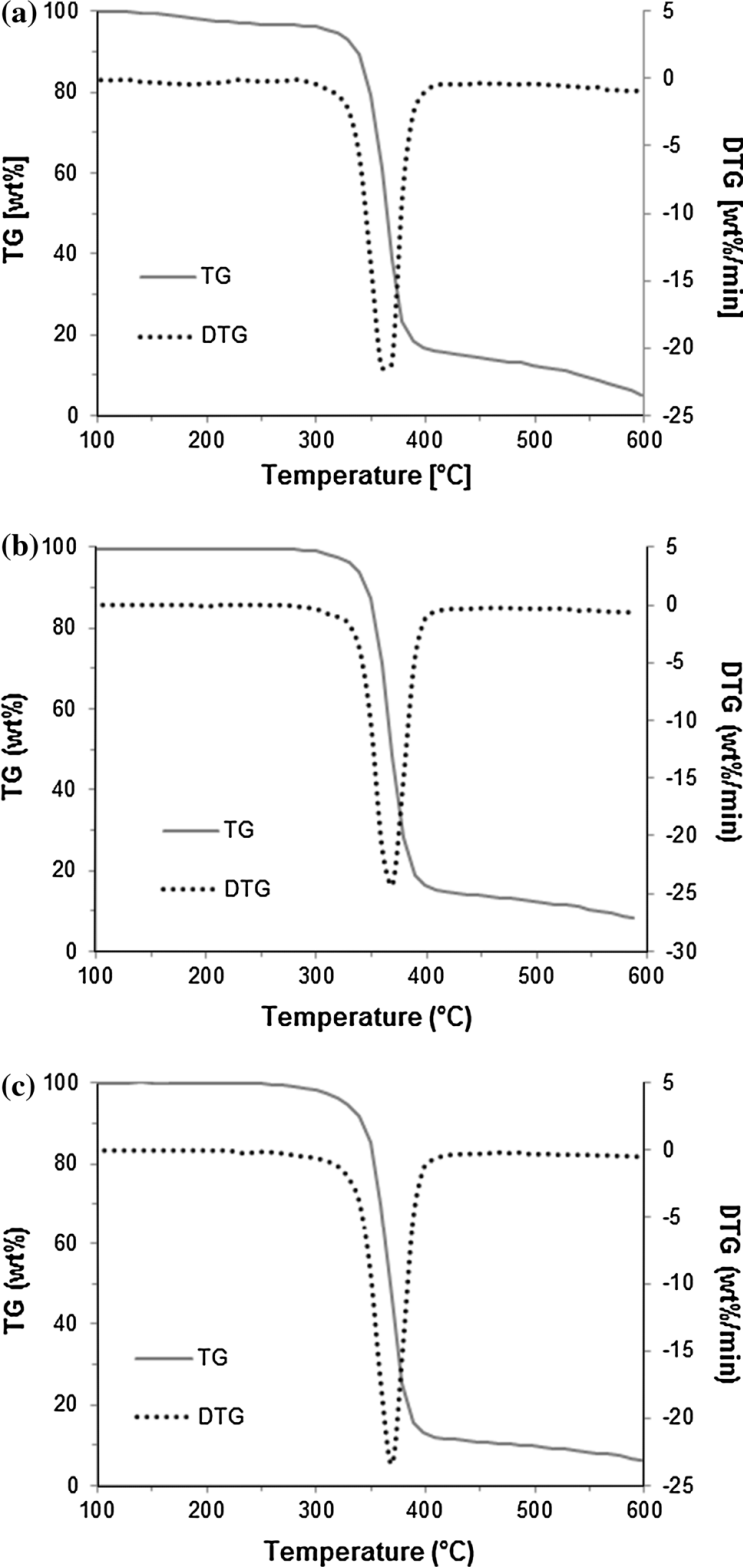
TGA and DTGA curves of CAB-17, CAB-35 and CAB-52

In Fig. 3, the TGA and the 1st derivative of the TGA curves show that the decomposition of the different CABs is very similar. For CAB-17, it starts around 320 °C while for CAB-35 and CAB-52 at 340 °C. The thermal degradation of cellulose esters consist of a series of degradation reactions. First, dehydration occurs below 100 °C, then the deacetylation and the debutyrylation take place at the beginning of the melting, and finally pyrolysis of cellulose skeleton takes place [15]. The DTG curves confirmed that there is only one main decomposition step for all CAB samples which is related to the degradation of the cellulose main chain. The maximum decomposition temperature was around 360–370 °C for all the samples. This implies that the difference in butyryl content does not have a significant effect on the thermal stability of the cellulose chain. At 600 °C all the material is decomposed. This temperature range is attributed to the thermal cleavage of the glycosyl units and scission of other C=O bonds via a free radical reaction [24].

### Transport simulations

**Fig. 4:**
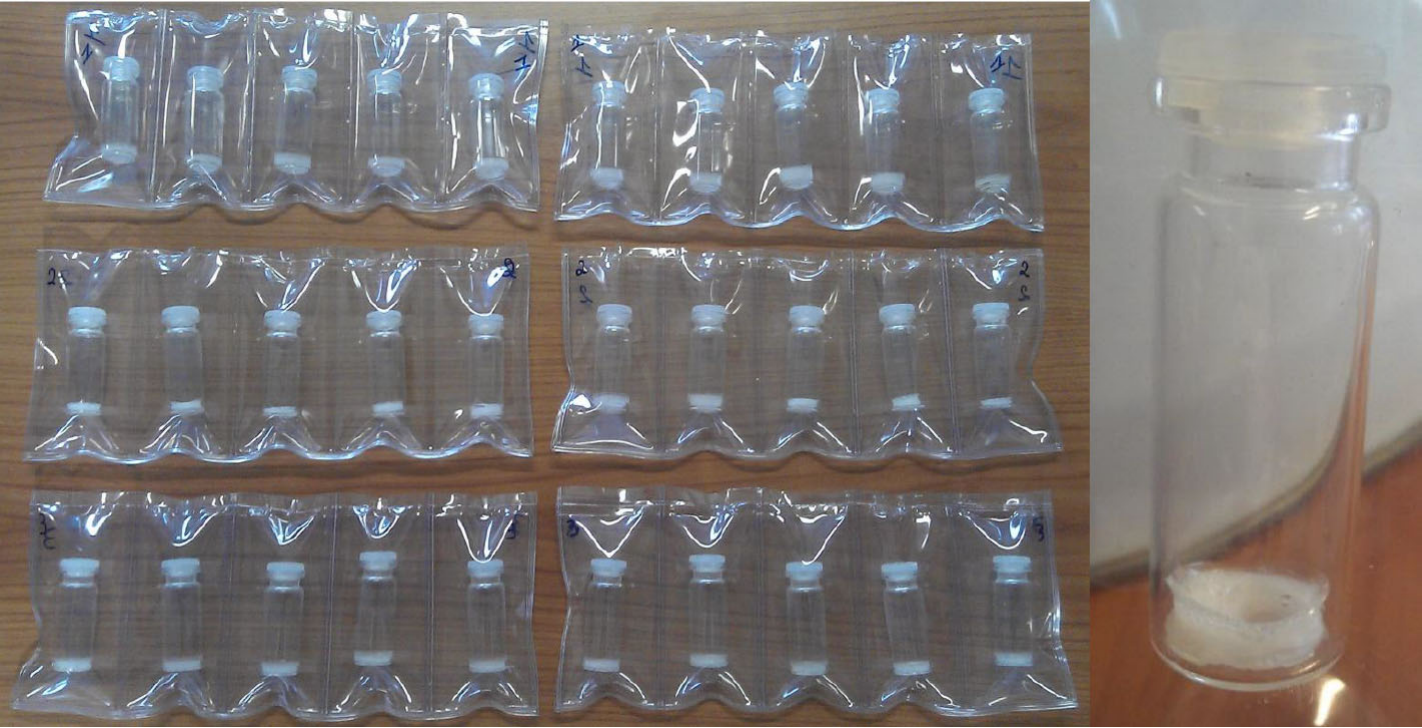
Samples after transport simulations incorporating Mg(NO_3_)_2_ salt

The LSD spikes are transported from the JRC-Geel to the customers; therefore transport simulations were carried out to see the impact of vibrations on the CAB matrix. In thin films, ab initio an internal stress exists due to the film formation; the inability of the film to shrink from evaporation of the solvent and adhere to the glass at the same time; temperature changing and relative humidity [25]. These stresses have an effect on the adhesion of the coating and could lead to delamination [26]. Moreover, film defects or local imperfections such as bubbles for instance can lead also to stress concentrations. When a stress is introduced for instance by vibrations, all the stress will concentrate at the imperfection which can result finally in formation of cracks. This can also cause delamination via crack initiation and propagation [27]. On the one hand, it is very difficult to predict the importance of the stress contributions to the physical deterioration of the CAB because the chemical degradation usually disguises the effect of physical ageing. On the other hand, one still has to take into account the physical ageing as well as it has an effect on the life time of the spike material. The transport by car and the simulation of truck and airplane showed no cracks or delamination of the test samples even if they had not adhered very well to the glass vials because of the incorporation of magnesium-nitrate in the cellulose matrix (see Fig. 4).

Thus, we can conclude that vibrations caused by the transport do not have an immediate effect on the integrity of the spike; however it could have a long term effect as the vibrations could create an additional internal stress in the matrix. Our experience with the transport of actual spikes is that flaking occurs very seldom directly after the arrival of the LSD spikes: our customers reported 1 or 2 flaking samples out of 800 units sold per year and this does not happen every year. These flaking phenomena can be related to some imperfections—mainly bubbles—in the thin layer, which together with the stress that accumulates during transport leads finally to the early delamination of the material.

### UV light irradiation

**Fig. 5:**
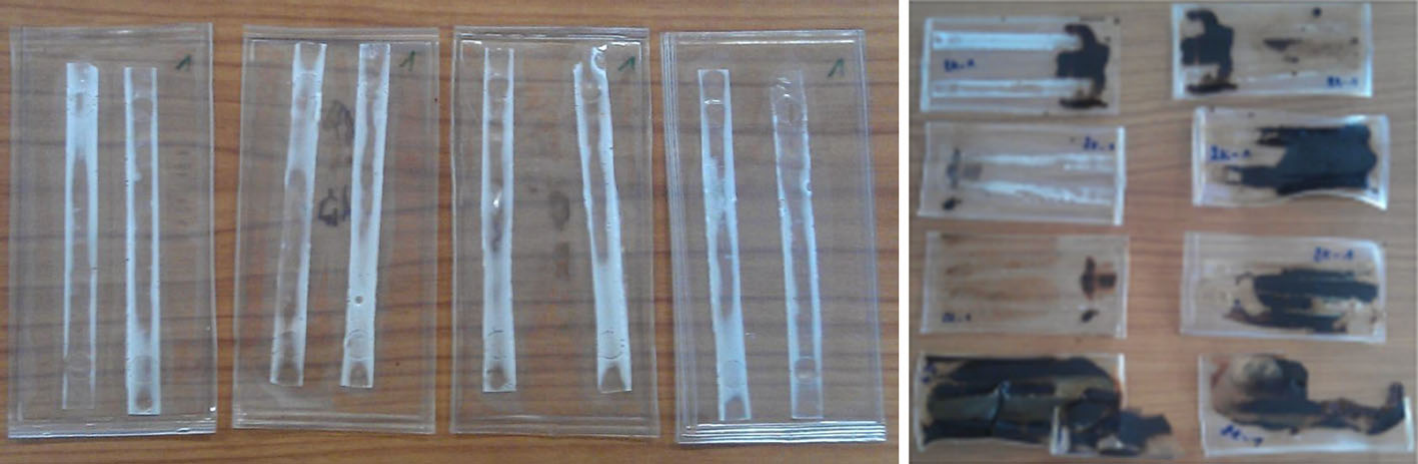
Tensile test samples of CAB-35 after 1000 (*left*) and 2000 h (*right*) of ageing in UV light

The xenon test is an accelerated ageing experiment meant to test the exterior durability, so to simulate outdoor weathering conditions like UV light, temperature, and humidity. In reality, the LSD spikes are never exposed to such harsh conditions; nevertheless the behaviour of the three types of CAB can be used to compare them and make a ranking for our application. Figure 5 shows the CAB-35 tensile samples after irradiation 1000 h (left) and 2000 h (right) in the Xenon test apparatus. As it can be seen, most of the samples after 1000 h of irradiation—equivalent to 1 year of normal ageing—were still intact and it was possible to perform the tensile and GPC tests. The samples that were exposed for 2000 h were black and decomposed and the sample holder PVC bags became brittle. Tensile tests on these samples were thus not feasible to perform. The molecular weight and the molecular weight distribution contribute significantly to the properties of the material. In Table 3, the different molecular weights from the GPC measurements are summarized. For CAB-17 and CAB-35, all the values significantly decrease after 2000 h of exposure, for CAB-52 the degradation was so severe that there was not enough material for the GPC analysis which can be related to the initially low *M*_n_ of CAB-52: 28 000 g mol^−1^ compared to 98 000 g mol^−1^ of CAB-17 and to 116 000 g mol^−1^ of CAB-35. After 1000 h of irradiation, the values are comparable or some of them are even a bit higher (e.g. *M*_w_ and *M*_Z_ of CAB-17 and CAB-52) than of the virgin ones. This can be explained by further polymerisation of the cellulose due to the effect of UV light [28]. However, this effect diminishes after 2000 h when the degradation process becomes dominant.

**Table 3:** Molecular weights from GPC measurements (only one sample was measured)

	*M*_n_ (g mol^−1^)	*M*_w_ (g mol^−1^)	*M*_z_ (g mol^−1^)	*M*_P_ (g mol^−1^)
CAB-17				
Virgin	98,340	202,420	364,050	171,200
1000 h	78,550	207,610	404,490	185,360
2000 h	16,480	41,140	85,890	29,330
CAB-35				
Virgin	115,940	250,140	479,630	479,630
1000 h	61,150	176,190	429,650	57,610
2000 h	22,270	118,750	389,630	42,270
CAB-52				
Virgin	27,990	64,020	116,100	55,900
1000 h	23,000	69,330	181,180	29,790
2000 h	NA	NA	NA	NA

**Fig. 6:**
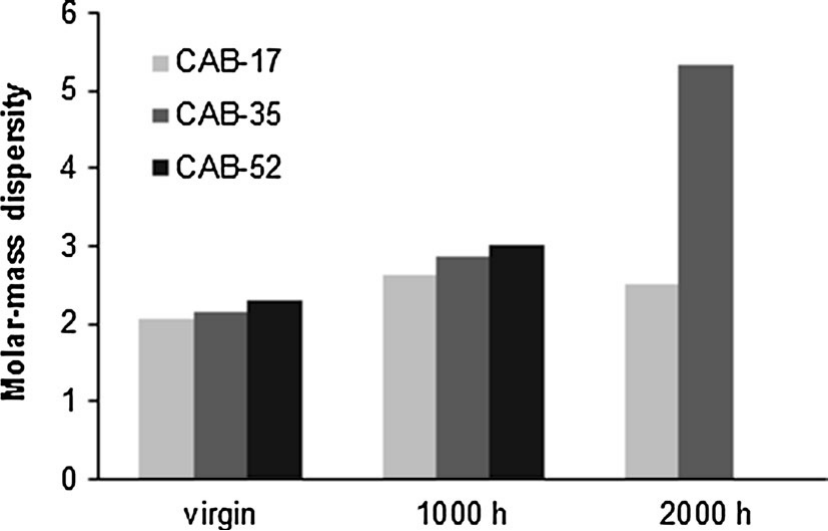
Molar mass dispersity of the three CAB samples—virgin and UV-aged samples

In Fig. 6, the molar mass dispersity values are shown. The values increase with longer exposure which means that there is a big variation in chain lengths within the polymer. When structural changes occur in a polymer due to degradation, the number-average molecular weight changes as well and this determines the mechanical properties of the material [29]. The mechanical properties such as the stress at break, the Young’s modulus and the strain at break with the standard deviation before and after 1000 h of UV ageing are summarised in Table 4. The first batch of samples of CAB-17 that were prepared for the tensile testing was placed in the Xenon test chamber. When attempting to prepare further batches, the thin film samples were deformed and curled thus it was not possible to perform tensile tests on them. Basically, CAB-17 is a more brittle material and this we have experienced during sample preparation. The reason why we could prepare the first batch successfully and the rest we could not remained unclear; therefore data are not available for the virgin samples of CAB-17. Despite that measurement data are missing on the tensile test we assume that CAB-17 has the higher tensile strength then CAB-35 and CAB-52 thanks to its low butyryl content and high molecular weight: 65,000 g mol^−1^.

**Table 4:** Tensile parameters with their standard deviations of the virgin and UV light aged samples after 1000 h

	Stress at break (N mm^−2^)	Modulus (MPa)	Strain at break (%)
CAB-17			
Virgin	NA	NA	NA
1000 h	12.7 ± 5.5	1591.5 ± 112.9	0.89 ± 0.25
CAB-35			
Virgin	9.8 ± 1.6	1010.6 ± 60.1	1.02 ± 0.18
1000 h	4.1 ± 0.6	588.4 ± 84.1	0.95 ± 0.18
CAB-52			
Virgin	2.3 ± 0.3	409.7 ± 97.5	0.65 ± 0.29
1000 h	2.3 ± 0.5	267.7 ± 82.8	1.01 ± 0.09

It can be observed that the modulus of the non-aged and of the UV exposed samples have a tendency to decrease to approximately 50 %: thanks to the ageing the mechanical strength reduces due to the chemical degradation of the polymer. Comparing the strain at break values it either does not change—for CAB-35 it is within the range of standard deviation—or in case of CAB-52 it has been even increased. This phenomenon can be explained with further polymerisation of the cellulose due to the effect of UV light. It can be stated that the higher the butyryl content, the lower the modulus and so the stiffness. This behaviour is linked to the properties of butyryl group as it causes rubber-like mechanical effects, thus gives more flexibility to the polymer. The stiffer the sample the less resistant to elastic deformation which could be accompanied with higher strain at the break and this was confirmed by our measurements.

### X-ray irradiation

**Fig. 7:**
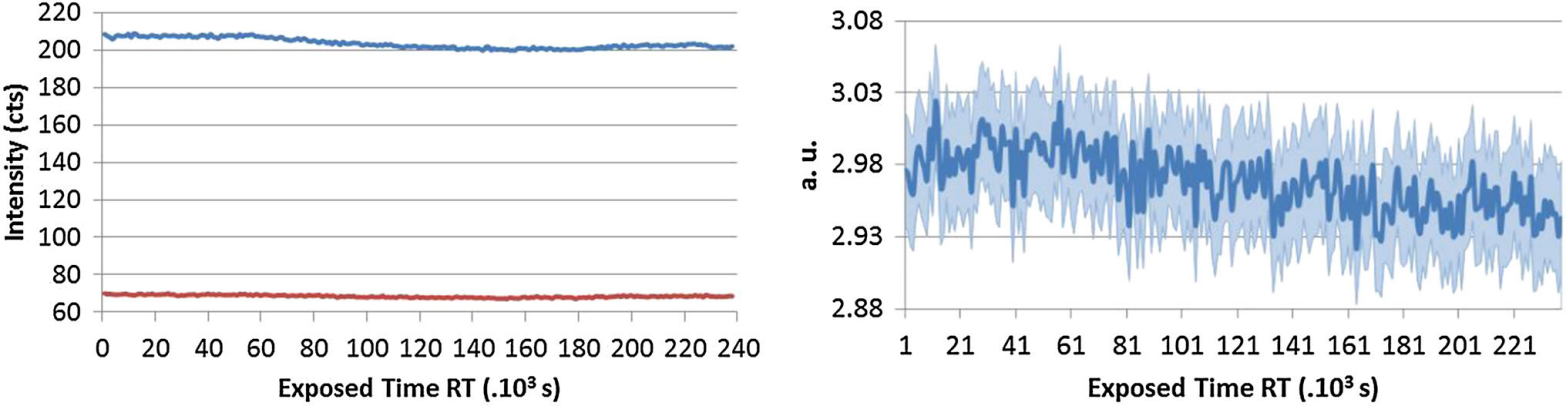
CAB-17 Normalized Compton (*blue* or *upper curve*) and Rayleigh (*red* or *lower curve*) intensity changes (*left*) Mass changes through Compton/Rayleigh with time (*right*). (Color figure online)

**Fig. 8:**
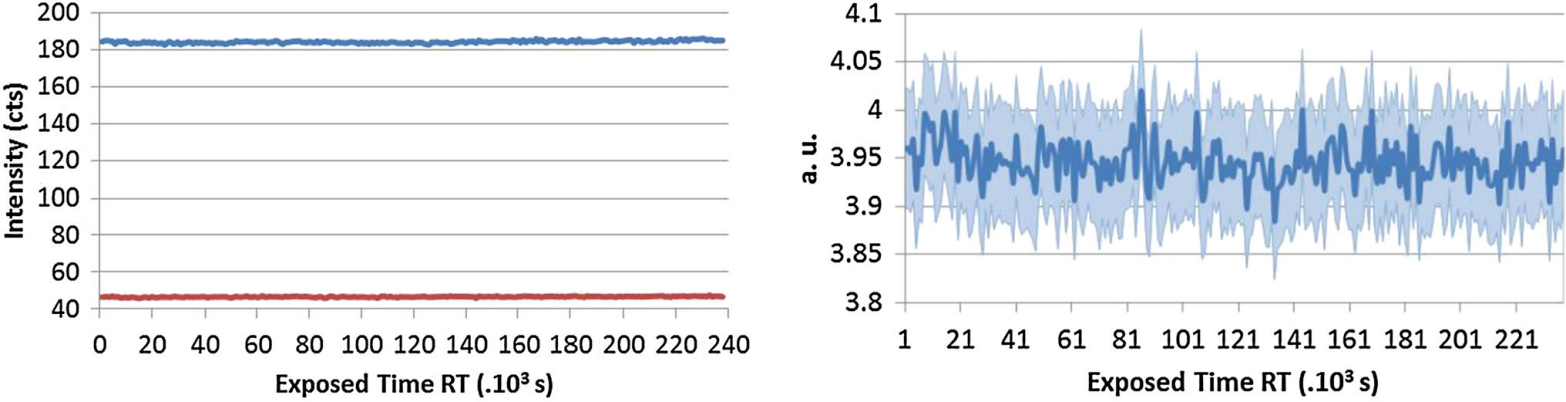
CAB-35 Normalized Compton (*blue* or *upper curve*) and Rayleigh (*red* or *lower curve*) intensity changes (*left*) Mass changes through Compton/Rayleigh with time (*right*). (Color figure online)

**Fig. 9:**
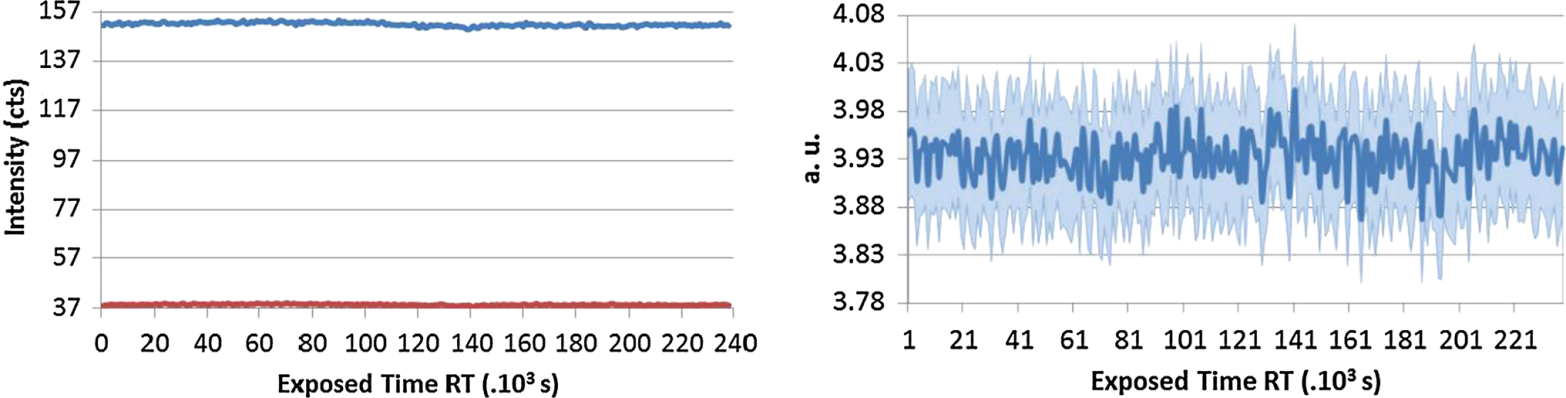
CAB-52 Compton (*blue* or *upper curve*) and Rayleigh (*red* or *lower curve*) intensity changes (*left*) Mass changes through Compton/Rayleigh with time (*right*). (Color figure online)

As the LSD spikes contain radioactive material, the absorption of the alpha and gamma radiation in the material itself has a major contribution to the deterioration of the CAB. The U and Pu are embedded in the cellulose matrix [30] and the charged alpha particles and gamma rays can cause scission of the polymer. The main activity contributors are: Pu-239 which is an alpha emitter and Pu-241 which is a beta emitter, 4 and 1.6 MBq, respectively. Ionizing radiation can either induce cross-linking within the cellulose chain [31] or can also result in degradation of cellulose based materials [16, 32] which depends on the absorbed dose, the type of radiation used and the state of matter as well. As it was not possible to investigate the original material with state of the art techniques in a glove box due to the U and Pu presence at the JRC-Geel, an irradiation experiment of the virgin CAB samples with X-rays was designed and carried out at the Ghent University. The interaction of the CAB with X-rays is different from the alpha particles; nevertheless the experiment still gives an indication of how the different types of CAB perform under exposure to ionizing radiation. There is evidence that irradiation of cellulose acetate (CA) with X-rays resulted in gas evolution—CO, CO_2_ and H_2_—and cracks and discoloration could be observed [33]. It was also reported that CAB-35 irradiated with gamma rays resulted in a loss of ester bonds and this effect increased with increasing dose rate [11]. The three types of CAB samples were therefore irradiated in a so-called micro-XRF device for 3 days [34]. The 3 days irradiation corresponded approximately to 3 years of storage of the CRM. The X-rays were monochromatic and the changes in the material were recorded by following the Compton and Rayleigh scattering (see Figs. 7, 8, 9).

**Fig. 10:**
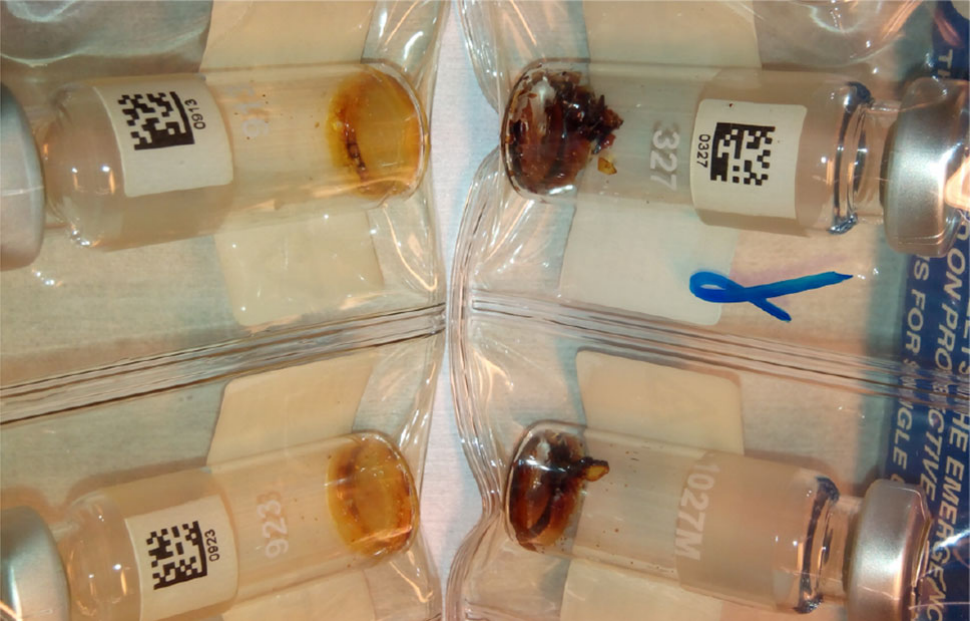
CAB-35 (*left*) and CAB-17 (*right*) appereance on the IRMM-1027m batch of LSD spikes after 5 years of storage

When some structural changes occur in the polymer the density changes as well. This provides a direct evidence for the degradation of the material. As it can be seen in Fig. 7, the Compton scattering intensity slightly decreases for CAB-17 after approximately 80,000 s irradiation which corresponds to about a year exposure. In the right side of Fig. 7 the Compton/Rayleigh fraction shows the irradiated mass (density × thickness) of the sample. As the polymer breaks down, it is assumed that the irradiated mass changes as well. For CAB-35 and CAB-52 (Fig. 8, 9), no changes in the local density can be detected thus there is no evidence for the polymer degradation under irradiation with X-rays. At the JRC-Geel, we have experience with CAB-35 applied to the batch IRMM-1027 m, a batch that was prepared in 2009. The spikes covered with CAB-35 properly stored in an upright position and which were not exposed to vibrations due to transport, stayed intact for 5 years (Fig. 10, left hand side) while the spikes covered with CAB-17 are already deteriorated and flaked off (Fig. 10, right hand side). This empirical experience already proved that for our specific application on LSD spikes, CAB with 35 wt% butyryl has a longer life time then CAB with 17 wt% butyryl substitution. Thus, this study carried out at the JRC-Geel is line with the experiments of X-ray irradiation confirming that CAB with higher butyryl content seems to be more resistant to ionizing radiation. The samples were so small that it was not possible to analyse them after the irradiation with the GPC technique to measure the molecular weights. As the long term stability of the IRMM-1027 LSD spike material is determined by the life time of the CAB, it is very crucial to find the most suitable butyryl substitution. Therefore, further empirical studies will be carried out blending CAB-17 and CAB-52 with CAB-35 and apply them on the U-Pu spikes.

## Conclusions

The long-term stability of CAB-17, CAB-35 and CAB-52 were investigated under different ageing conditions in order to select the most robust and chemically resistant CAB for the series of IRMM-1027 LSD spikes. Thin film samples were prepared without U and Pu and characterized before and after ageing with TGA, DSC, GPC, tensile test and via Compton and Rayleigh scattering. The samples were aged by transport, UV light, humidity, temperature and X-rays. CAB-17 showed better thermal and mechanical stability while CAB-35 and CAB-52 were more resistant against weathering and ionizing radiation. CAB-35, which was proven at the JRC-Geel to have a long-term stability of over 5 years, therefore it was chosen as an organic matrix for the production of LSD spikes and the validity of the certificate is issued for 3 years. For the future, the results of this study give an indication that a slightly different cellulose acetate versus butyryl content together with fine tuning of the chemical procedure which will possibly allow us to prolong the validity of our certificates even beyond 3 years.
